# Optimizing coronary CT angiography in patients with delayed contrast arrival using a free-breathing, high-threshold bolus-tracking protocol

**DOI:** 10.1186/s13244-026-02368-4

**Published:** 2026-08-15

**Authors:** Yan Zhang, Ying Wang, Jing Li, Yixuan Zou, Aihui Di, Ning Lang, Huishu Yuan

**Affiliations:** 1 Institute of Large-Scale Scientific Facility, Beihang University, Beijing, China; 2 Department of Radiology, Peking University Third Hospital, Beijing, China; 3 United Imaging Healthcare, Shanghai, China; 4 State Key Laboratory of Vascular Homeostasis and Remodeling, Peking University Third Hospital, Beijing, China

**Keywords:** Coronary computed tomography angiography, Contrast media, Bolus tracking, Image quality, Coronary artery disease

## Abstract

**Objective:**

To investigate the performance of a free-breathing coronary computed tomography angiography (CCTA) protocol using a high-threshold bolus-tracking strategy in improving coronary enhancement and diagnostic visualization in patients with delayed contrast arrival.

**Materials and methods:**

In this prospective study, 159 patients undergoing CCTA were randomized to either a conventional bolus-tracking protocol (breath-hold, 100 HU trigger threshold, 6.8 s delay) or a modified high-threshold protocol (free-breathing, 300 HU trigger threshold, 2 s delay). Patients were stratified according to contrast arrival time (*T*_arr_) into normal and delayed contrast arrival groups using a predefined operational cutoff (15 s), resulting in four subgroups. Coronary attenuation, enhancement uniformity as assessed by the coefficient of variation (COV), enhancement phase distribution, and image quality scores were compared among groups.

**Results:**

Under the conventional protocol, patients with delayed contrast arrival demonstrated reduced enhancement uniformity and a higher proportion of late-phase acquisitions. Compared with the conventional protocol, the modified protocol significantly increased coronary attenuation and improved enhancement uniformity (COV: 11.28% vs 16.73%, *p* < 0.001), as well as the proportion of segments with excellent image quality in patients with delayed contrast arrival (94.2% vs 86.8%, *p* = 0.004). In this subgroup, the modified protocol yielded more optimal-phase scans (19.4% vs 9.4%) and fewer late-phase acquisitions (32.3% vs 65.6%). Visual quality scores and quantitative metrics were consistently higher with the modified protocol across all major coronary arteries (all *p* < 0.001).

**Conclusion:**

The free-breathing, high-threshold bolus-tracking CCTA protocol improves enhancement uniformity and facilitates optimal scan-phase acquisition in patients with delayed contrast arrival, providing a more reliable and patient-tolerant alternative to conventional breath-hold protocols.

**Key Points:**

**Question** Can a free-breathing high-threshold bolus-tracking strategy improve coronary enhancement timing and image quality in CCTA patients with delayed contrast arrival?

**Findings** The modified protocol increased optimal-phase acquisitions, improved coronary enhancement uniformity, and maintained diagnostic image quality in patients with delayed contrast transit.

**Critical relevance statement** A free-breathing high-threshold bolus-tracking strategy may improve acquisition robustness and enhancement consistency in patients with unpredictable contrast transit, potentially expanding the clinical applicability of CCTA in individuals with impaired breath-hold capacity or delayed circulation dynamics.

**Graphical Abstract:**

**Figure:**
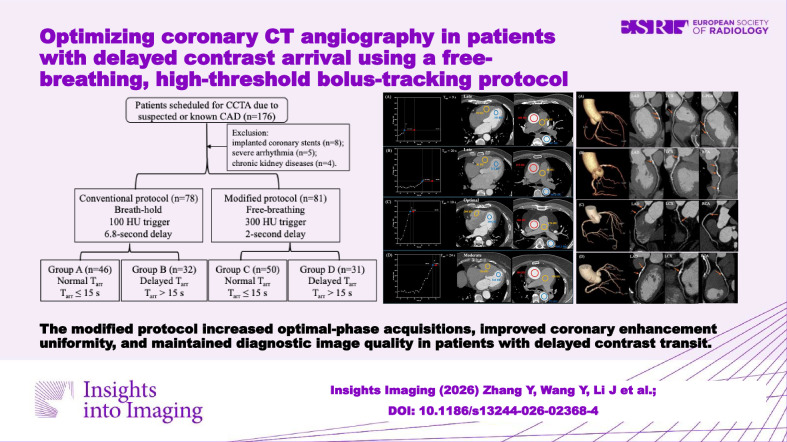

## Introduction

Coronary computed tomography angiography (CCTA) has become a pivotal non-invasive imaging modality for diagnosing and managing coronary artery disease (CAD) [1, 2], which provides high diagnostic accuracy and prognostic value [3, 4]. However, the diagnostic performance of CCTA relies heavily on accurate synchronization between peak coronary artery enhancement and image acquisition. Factors such as cardiac output, body habitus, contrast injection protocol, and breathing status can all influence enhancement patterns [5].

In CCTA, the contrast arrival time (*T*_arr_) is defined as the interval from the initiation of intravenous contrast injection to the moment when the attenuation in a predefined monitoring region, typically the ascending aorta (AAO), reaches a predefined trigger threshold [6, 7]. This parameter is critical for accurately timing the image acquisition to coincide with peak coronary enhancement. However, *T*_arr_ is influenced by a combination of cardiac and extracardiac factors, including reduced cardiac output, heart failure, vascular conditions such as atherosclerosis, pulmonary circulation status, and patient body size [6–10]. Consequently, in routine clinical practice, a subset of patients undergoing CCTA exhibits a prolonged *T*_arr_ during bolus tracking even in the absence of overt heart failure. For these individuals, it is difficult to predict *T*_arr_ before the examination. It challenges the application of conventional CCTA protocols.

Conventional CCTA protocols typically employ breath-hold acquisitions combined with bolus tracking using a fixed aortic trigger threshold and preset post-trigger delays [11–14]. These strategies assume relatively uniform contrast arrival time and respiratory conditions across all patients. Under delayed contrast arrival conditions, however, fixed-delay approaches are prone to mistimed acquisition, resulting in suboptimal coronary opacification or increased attenuation heterogeneity [11, 15]. Although using the test-bolus techniques may improve temporal accuracy, they require additional contrast administration (10–20 mL) and radiation exposure [2]. Moreover, enforced breath-holding may perturb venous return and respiratory mechanics, further compromising image quality in susceptible patients [16–18].

To address these two gaps, we developed a modified CCTA acquisition strategy designed to improve coronary imaging performance in patients with delayed contrast arrival. This approach integrates a higher bolus-tracking trigger threshold (300 HU) with an ultra-short post-trigger delay (2 s) to initiate image acquisition immediately upon reaching diagnostically relevant enhancement levels. This is designed to eliminate reliance on the prediction of individual circulation time, thereby directly mitigating mistimed acquisitions due to variable contrast arrival. In addition, free-breathing acquisition is implemented to improve scan tolerance and consistency, because patients with delayed contrast arrival, as aforementioned, often have diminished cardiopulmonary reserve and cannot maintain breath-holds reliably. The present prospective study evaluates the performance of this strategy across varying contrast arrival conditions, with a particular focus on enhancement uniformity, scan-phase distribution, and image quality.

## Materials and methods

### Participants enrollment and grouping

This prospective study was approved by the Medical Ethics Committee of Peking University Third Hospital (Approval No. [(2022)-420-01]). All participants provided written informed consent before undergoing CT examination. Patients scheduled for CCTA from February 2022 to October 2025 due to suspected or known CAD were consecutively enrolled. Patients with implanted coronary stents were excluded because metal artifacts may be involved, which is important but a separate issue beyond dose and vessel enhancement as considered in the present study. Patients with severe arrhythmia (defined as persistent atrial fibrillation or frequent premature beats), chronic kidney disease (defined as glomerular filtration rate < 30 mL/min/1.73 m^2^), or clinically diagnosed heart failure (New York Heart Association Class IV) were also excluded.

**Fig. 1:**
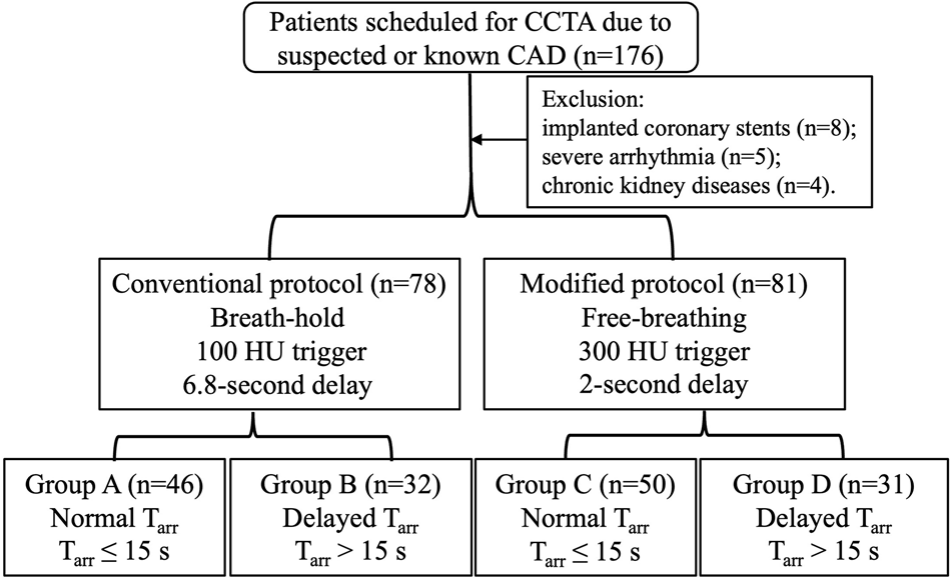
Flowchart of study design. CAD, coronary artery disease; CCTA, coronary computed tomography angiography; *T*_arr_, contrast arrival time

Patients were prospectively assigned to one of two scanning protocols using a computer-generated randomization sequence in a 1:1 ratio. This was performed by an independent research coordinator who was not involved in image acquisition or analysis. Participants were randomized to either (1) a conventional breath-hold protocol employing bolus tracking with a trigger threshold of 100 HU and a fixed post-trigger delay of 6.8 s, or (2) a modified free-breathing protocol using a higher trigger threshold of 300 HU combined with an ultra-short post-trigger delay of 2 s (Fig. 1).

Contrast arrival characteristics were subsequently assessed using *T*_arr_, defined as the interval between contrast injection and the time point at which attenuation in the AAO exceeded 50 HU during bolus tracking [5]. *T*_arr_ was used solely as a CT-derived descriptor of contrast transit timing rather than as an indicator of cardiac function. Based on an operational cutoff reported in prior CCTA timing studies, participants were categorized into normal contrast arrival (*T*_arr_ ≤ 15 s) and delayed contrast arrival (*T*_arr_ > 15 s) groups [5, 8]. Accordingly, four subgroups were defined for analysis: Group A, conventional–normal *T*_arr_; Group B, conventional–delayed *T*_arr_; group C, modified–normal *T*_arr_; and group D, modified–delayed *T*_arr_.

### CT scanning protocol and image reconstruction

All CT examinations were performed on a 320-row CT scanner (uCT 960+; United Imaging Healthcare) with z-coverage of 120, 140 or 160 mm dependent on patient heart size, and secondary reconstruction with artificial intelligence (AI)-based motion correction algorithm (CardioCapture; United Imaging Healthcare) to perform CCTA with the following characteristics: tube voltage 100 kVp; tube current 200 mAs; gantry rotation time 0.25 s. Prospective electrocardiography (ECG)-triggered acquisition was performed within a single cardiac cycle. The acquisition window was set at 30%–80% of the R–R interval to improve the likelihood of capturing an optimal motion-free reconstruction phase in patients with variable heart rates and delayed contrast transit. The optimal diagnostic phase was initially determined automatically by the reconstruction software based on the ECG signal (ePhase; United Imaging Healthcare) and subsequently confirmed by experienced readers during image interpretation. The scan range covered the entire heart, from 1 cm below the carina up to the diaphragm. As in the majority of routine cases, beta-blockers were not used.

All patients received a 45 mL bolus of contrast medium (350 mg/mL, Ioversol, Bayer) with 12 s injection time via an antecubital vein at an infusion rate of 5 mL/s, followed by a 40 mL saline flush at the same rate. Contrast injection was performed using a dual-head power injector (ANT200200L; Antmed) through a 20-gauge intravenous cannula, with standard clinical extension tubing. Injection parameters were kept constant across all participants to minimize variability in bolus geometry and contrast transit characteristics.

Bolus tracking was employed in all examinations, with the region of interest placed in the descending aorta at a level 1 cm below the tracheal bifurcation. For the conventional breath-hold protocol (Groups A and B), image acquisition was triggered when the attenuation reached 100 HU, followed by a fixed post-trigger delay of 6.8 s. Patients were instructed to hold their breath after a normal inspiration during the delay period, and CCTA acquisition was performed during breath-holding.

For the modified free-breathing protocol (Groups C and D), a higher trigger threshold of 300 HU was applied in combination with an ultra-short post-trigger delay of 2 s, and image acquisition was performed without interrupting respiration. For the modified free-breathing protocol (Groups C and D), patients received standardized respiratory instructions before image acquisition. Specifically, they were instructed to maintain gentle, regular tidal breathing throughout the examination while avoiding deep inspiration, breath-holding, coughing, or irregular respiratory maneuvers. To improve reproducibility, patients underwent a brief pre-scan breathing rehearsal under technologist guidance to familiarize them with the required breathing pattern. During acquisition, respiratory motion was visually monitored by the technologist, and scanning was initiated only when a stable and consistent breathing pattern was observed. Because the acquisition duration was shorter than 1 s in most patients, respiratory displacement during data acquisition was expected to remain minimal.

The higher trigger threshold was selected to ensure that image acquisition was initiated at a diagnostically relevant enhancement level, thereby reducing sensitivity to delayed or heterogeneous contrast transit. The 2-s post-trigger delay represents the shortest delay technically achievable on the scanner and was chosen to minimize the temporal gap between reaching the enhancement threshold and data acquisition, reducing the risk of late-phase scanning under delayed contrast arrival conditions.

All CT images were reconstructed with a routinely available hybrid iterative algorithm (Karl 3D; United Imaging Healthcare; Shanghai; China), with a 512 × 512 pixels matrix, 0.5 mm slice thickness, and 0.5 mm slice interval. The data were then transferred to the clinical post-processing workstation (uWS-CT: R006; United Imaging Healthcare; Shanghai; China) to generate volume rendering (VR), maximum intensity projection (MIP), and curved planar reconstruction (CPR) images for each of the following analyses.

The scan time, volume CT dose index (CTDI_vol_), and dose length product (DLP) for each patient were retrieved from the dose reports stored in the picture archiving and communication system. The effective dose (ED) was calculated by multiplying the DLP by a standardized chest conversion coefficient (*k* = 0.014 mSv/mGy·cm) [19]. Radiation dose analysis was limited to the diagnostic acquisition phase.

### Subjective image quality assessment

Two radiologists, one with 10 years and the other with 15 years of experience in CCTA interpretation, independently performed the visual assessment of coronary artery imaging. Both readers were blinded to the angiographic and clinical information, and cases were presented in random order to avoid bias related to the scanning protocol. The evaluation was performed for vessels with a diameter > 1.5 mm on MIP reconstruction images according to the 18-segment classification system, which included proximal, mid, and distal coronary branches and thereby allowed indirect assessment of distal vessel visualization. All segments were graded using a 4-point Likert scale [20], reflecting visual quality in terms of contrast enhancement, vessel visualization, and noise appearance (score 4 = excellent; score 3 = acceptable; score 2 = inferior, non-diagnostic; score 1 = non-acceptable). A score ≥ 3 was considered acceptable for diagnosis. To account for intra-patient correlation among coronary segments, an additional patient-level analysis was performed using the mean subjective segment score per patient for group comparisons. Disagreements were resolved by consensus between the same two radiologists after joint review.

### Quantitative measurement of CCTA image

**Fig. 2:**
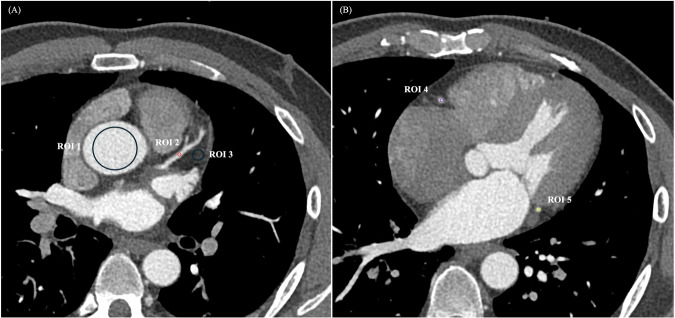
Placement of regions of interest (ROIs) for quantitative attenuation measurements. ROI 1–5 represent the AAO, LAD, pericardial fat, RCA, and LCX artery, respectively

One radiologist with over 15 years of experience in cardiovascular imaging manually sketched the ROIs to perform the quantitative assessment of coronary contrast enhancement (Fig. 2). Attenuation values and noise were measured on AAO (ROI 1), the right coronary artery (RCA, ROI 4), left anterior descending (LAD, ROI 2) artery, the left circumflex (LCX, ROI 5) artery, as well as the adjacent pericardial fat (ROI 3). The ROI size on the pericardial fat was 12 mm^2^. Other ROIs were made as large as possible according to the lumen of the vessel (1.2–1.7 mm^2^), away from the wall and any possible calcification or plaque. The noise was defined as the standard deviation (SD, HU) of CT attenuation within the ROI [20]. The signal-to-noise ratio (SNR) and contrast-to-noise ratio (CNR) were calculated using SNR = μ_artery_/SD_artery_; CNR = (μ_artery_ − μ_fat_) / SD_fat_, where μ stands for the CT attenuation within the ROI.

### Enhancement uniformity analysis

**Fig. 3:**
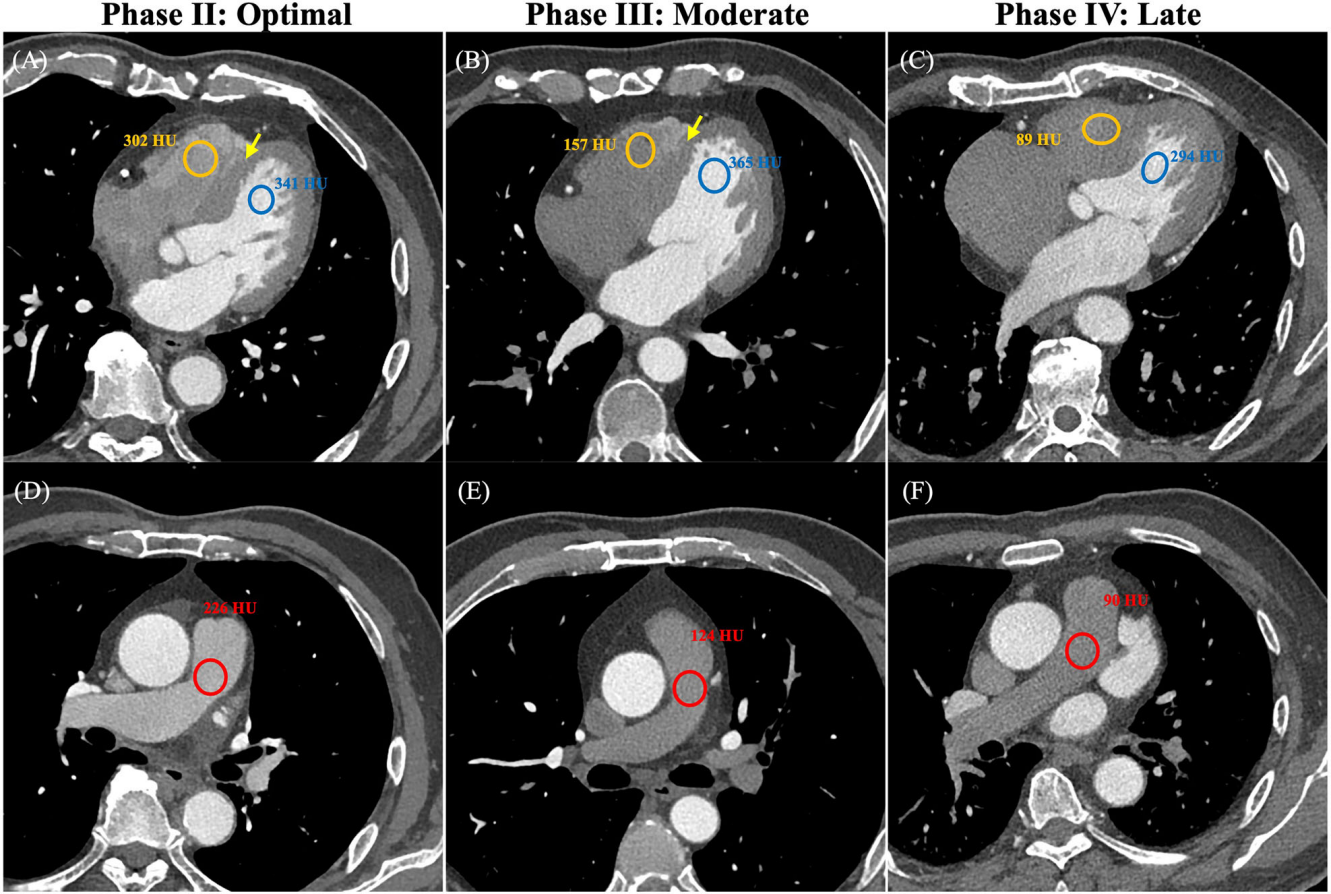
Representative coronary CTA images illustrating three contrast enhancement categories. **A**–**D** Phase II: optimal, marked enhancement of the LV (blue circle), with moderate opacification of the RV (orange circle) and PA (red circle); the IS is clearly visualized (yellow arrow). **B**–**E** Phase III: moderate, predominant enhancement of the LV (blue circle); RV (orange circle) and PA (red circle) show mild enhancement; IS is partially preserved (yellow arrow). **C**–**F** Phase IV: late, only the LV shows enhancement (blue circle); no visible enhancement in the RV (orange circle) and PA (red circle); the IS is poorly visualized

To evaluate contrast enhancement uniformity, the coefficient of variation (COV) of attenuation values in the AAO and coronary arteries was calculated at the subgroup level to reflect inter-patient variability in contrast enhancement. Specifically, COV was defined as COV = (SD/μ) × 100%, where SD represents the SD of attenuation values (HU) across patients within the same subgroup for each individual vessel, and μ represents the corresponding mean attenuation value of that vessel within the subgroup. Mean attenuation values represented the overall enhancement magnitude, while COV quantified enhancement heterogeneity [19, 21]. Circular ROIs were manually placed at specific anatomical levels, including the right ventricle (RV) and left ventricle (LV) at the mitral valve level, the interventricular septum (IS), and the pulmonary artery (PA). Additionally, the enhancement phase was determined according to the anatomical location of predominant contrast opacification, based on relative attenuation among the IS, RV, LV, and PA. According to predefined criteria (Table 1 and Fig. 3), scans were categorized into four phases reflecting progressive contrast transit from the right heart to the coronary circulation [21].

**Table 1:** Criteria for enhancement phase classification in CCTA

Phase	Description
Phase I: early	Complete visualization of the IS; right ventricular attenuation greater than left ventricular attenuation; PA attenuation > 250 HU.
Phase II: optimal	Complete visualization of the IS; left ventricular attenuation higher than right ventricular attenuation (difference ≥ 50 HU and < 150 HU); PA with moderate enhancement (≥ 150 HU and < 250 HU).
Phase III: moderate	Interventricular septal contour visible; left ventricular attenuation higher than right ventricular attenuation (difference ≥ 150 HU and ≤ 250 HU); PA with mild enhancement (≥ 100 HU and < 150 HU).
Phase IV: late	IS not visible; marked left ventricular enhancement with absent right ventricular enhancement; PA attenuation < 100 HU.

### Statistical analysis

All statistical analyses were performed using SPSS version 26.0 (IBM Corp., Armonk, NY, USA). Normality of continuous variables was assessed using the Shapiro–Wilk test. Data with normal distribution were presented as mean ± SD, while non-normally distributed variables were reported as median and interquartile range (IQR). Categorical variables were expressed as frequencies and percentages [*n* (%)]. Categorical data were compared using the Chi-square test or Fisher’s exact test, as appropriate. For baseline characteristics, group comparisons were conducted using one-way analysis of variance (ANOVA) for normally distributed variables and the Kruskal–Wallis *H*-test for non-normally distributed variables, followed by post hoc pairwise comparisons with Bonferroni (ANOVA) or Dunn’s test (Kruskal–Wallis) where appropriate. For two-group comparisons, independent-samples *t*-tests were used for normally distributed data, and Mann–Whitney *U*-tests for skewed data. The COV for the AAO and coronary arteries was used to assess enhancement uniformity, and Levene’s test was applied to evaluate differences in variance (dispersion) between groups. Interobserver agreement for visual grading was assessed using kappa statistics (κ), interpreted as follows: ≥ 0.81 = excellent, 0.61–0.80 = substantial, 0.41–0.60 = moderate, 0.21–0.40 = fair, and < 0.20 = poor. A two-tailed *p*-value < 0.05 was considered statistically significant.

## Results

### Patient characteristics and radiation dose

A total of 159 patients were included and stratified into four groups according to contrast arrival timing and scanning protocol (Table 2). There were no significant differences among the groups in terms of age, sex distribution, body mass index (BMI), cardiovascular risk factors, coronary dominance, or stenosis severity (all *p* > 0.05). The delayed contrast arrival groups exhibited approx. 5 s longer *T*_arr_ compared to the normal contrast arrival groups, reflecting heterogeneous contrast transit timing across the cohort. Scan duration tended to be slightly longer under delayed contrast arrival conditions with the conventional breath-hold protocol (Group B conventional–delayed *T*_arr_ 0.98 ± 0.21 s vs Group A conventional–normal *T*_arr_ 0.92 ± 0.08 s; *p* = 0.080). Radiation dose, assessed as ED, was higher in patients with delayed contrast arrival when the conventional protocol was used. Group B conventional–delayed *T*_arr_ exhibited the highest ED (5.51 ± 1.39 mSv), which was significantly higher than that of Group A conventional–normal *T*_arr_ (4.83 ± 1.04 mSv, *p* = 0.015). No statistically significant difference in ED was observed between Group D and Group A (*p* = 0.182). In contrast, among patients with delayed contrast arrival, application of the modified free-breathing protocol (Group D) resulted in a 7.4% reduction in ED compared with Group B conventional–delayed *T*_arr_ (*p* = 0.115), despite similar contrast arrival timing.

**Table 2:** Summary of the patient characteristics and radiation dose

Characteristics	Group A (conventional–normal *T*_arr_) *n* = 46	Group B (conventional–delayed *T*_arr_) *n* = 32	Group C (modified–normal *T*_arr_) *n* = 50	Group D (modified–delayed *T*_arr_) *n* = 31	*p*-value
Triggering threshold (HU)	100	100	300	300	–
Delay time (s)	6.8	6.8	2	2	–
Male, *n* (%)	44 (96%)	31 (97%)	48 (96%)	30 (97%)	1.000
Age (years)	51.98 ± 11.87	53.31 ± 10.75	54.12 ± 10.37	58.32 ± 10.18	0.090
BMI (kg/m^2^)	29.51 ± 2.51	29.49 ± 3.74	29.09 ± 2.74	28.73 ± 2.07	0.604
Contrast material arrival time (*T*_arr_, s)	11.85 ± 1.79	17.22 ± 1.34^a^	12.68 ± 2.07	16.90 ± 1.21^b^	0.001
Risk factors					
Hypertension, *n* (%)	28 (60.87%)	18 (56.25%)	31 (62.00%)	24 (77.42%)	0.320
Hyperlipidemia, *n* (%)	22 (47.83%)	14 (43.75%)	22 (44.00%)	14 (45.16%)	0.980
Diabetes, *n* (%)	10 (21.74%)	9 (28.13%)	11 (22.00%)	10 (32.26%)	0.675
Current or past smoking, *n* (%)	6 (13.04%)	0 (0%)	7 (14.00%)	5 (16.13%)	0.456
Family history of CAD, *n* (%)	18 (39.13%)	12 (37.50%)	14 (28.00%)	12 (38.71%)	0.639
Coronary dominance					
Right-sided, *n* (%)	45 (97.83%)	26 (81.25%)	42 (84.00%)	28 (90.32%)	0.093
Left-sided, *n* (%)	0 (0%)	2 (6.25%)	6 (12.00%)	2 (6.45%)	–
Codominant, *n* (%)	1 (2.17%)	4 (12.50%)	2 (4.00%)	1 (3.23%)	–
Stenosis severity					
RCA ≥ 50%, *n* (%)	6 (13.04%)	9 (28.13%)	14 (28.00%)	8 (25.81%)	0.278
LAD ≥ 50%, *n* (%)	11 (23.91%)	10 (31.25%)	15 (30.00%)	7 (22.58%)	0.789
LCX ≥ 50%, *n* (%)	7 (15.22%)	7 (21.88%)	8 (16%)	6 (19.35%)	0.865
Myocardial bridge, *n* (%)	7 (15.22%)	5 (15.63%)	18 (36.00%)^a^	7 (22.58%)	0.064
Scan characteristics					
Heart rate during scan (bpm)	62.69 ± 6.98	59.33 ± 7.91	64.08 ± 7.26	62.16 ± 12.06	0.104
Heart rate variation (bpm)^*^	12.02 ± 5.63	15.97 ± 15.14	6.66 ± 6.78^a^	11.23 ± 16.49^b^	0.003
Scan time (s)	0.92 ± 0.08	0.98 ± 0.21^b^	0.90 ± 0.09	0.92 ± 0.11	0.038
Radiation dose					
CTDI_vol_ (mGy)	23.08 ± 3.98	25.63 ± 6.96	24.14 ± 2.69	24.94 ± 2.96	0.059
DLP (mGy·cm)	344.67 ± 74.60	393.78 ± 99.40^a^	348.73 ± 47.98	373.28 ± 51.31^b^	0.008
ED (mSv)	4.83 ± 1.04	5.51 ± 1.39^a^	4.96 ± 0.74	5.13 ± 0.56	0.018

*BMI* body mass index, *CAD* coronary artery disease, *HU* Hounsfield units, *RCA* right coronary artery, *LAD* left anterior descending artery, *LCX* left circumflex artery, *CTDI*_vol_ volume CT dose index, *DLP* dose-length product, *ED* effective dose, *bpm* beats per minute

^*^Heart rate variability (HRV, bpm) was defined as the absolute difference between the maximum and minimum heart rates recorded during the CT acquisition period (HR_max_–HR_min_)

^a^Means this parameter was significantly different from that in Group A

^b^Means this parameter was significantly different from that in Group C

### Subjective image quality results

**Fig. 4:**
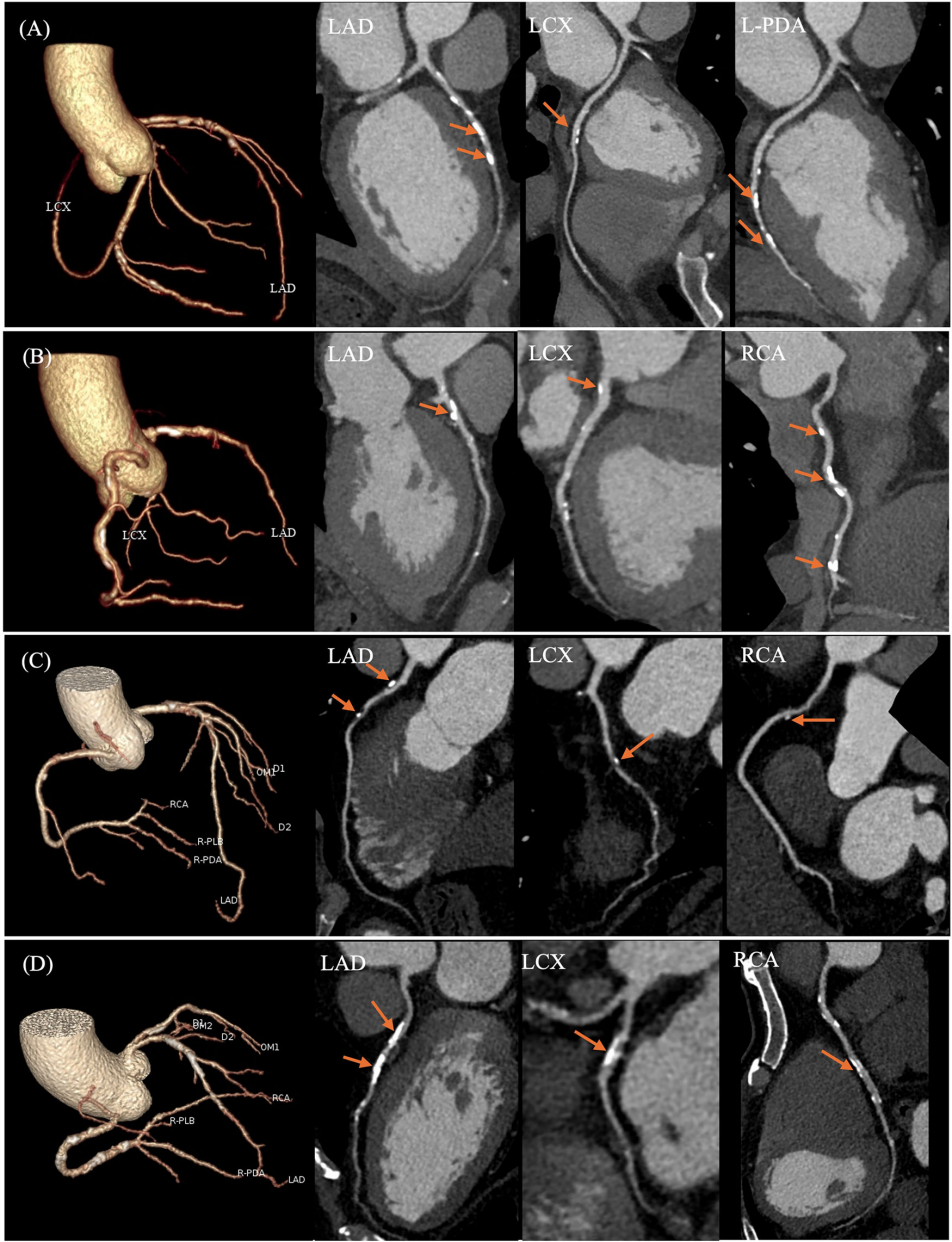
Coronary CTA images from matched cases across the four study groups. **A** VR and CPR image from a 50-year-old man (heart rate: 68 bpm; BMI: 30 kg/m²) in Group A (conventional–normal *T*_arr_), showing mixed plaque with moderate stenosis in the LAD artery, calcified plaque with mild stenosis in the LCX artery, and mixed plaque with severe stenosis in the left posterior descending artery (L-PDA). **B** VR and CPR images from a 63-year-old man (heart rate: 58 bpm; BMI: 29 kg/m²) in Group B (conventional–delayed *T*_arr_), showing mixed and calcified plaques with moderate stenosis in the LAD, calcified plaque with moderate stenosis in the LCX, and mixed plaque with severe stenosis in the RCA. **C** VR and CPR images from a 50-year-old man (heart rate: 60 bpm; BMI: 30 kg/m²) in Group C (modified–normal *T*_arr_), demonstrating mixed plaque with severe stenosis in both the LAD and LCX, and mixed plaque with moderate stenosis in the RCA. **D** VR and CPR images from a 66-year-old man (heart rate: 59 bpm; BMI: 28 kg/m²) in Group D (modified–delayed *T*_arr_), showing mixed and calcified plaques with severe stenosis involving the LAD, LCX, and RCA. Arrows indicate the locations of coronary plaques in each image

Subjective image quality assessment showed significant differences in coronary visualization across groups (Table 3). No coronary artery segment in any image was scored as less than 3. The proportion of segments rated as excellent (score 4) was highest in Group A conventional-normal *T*_arr_ (98.11%) and lowest in Group B conventional–delayed *T*_arr_ (86.83%). Group D modified-delayed *T*_arr_ showed marked improvement compared to Group B conventional–delayed *T*_arr_ (94.24% vs 86.83%, *p* < 0.001), indicating the benefit of the optimized protocol. Among major coronary arteries, Group B conventional–delayed *T*_arr_ had significantly lower mean scores than Group A conventional-normal *T*_arr_, including the LAD (3.91 vs 3.99), LCX (3.81 vs 3.97), and RCA (3.86 vs 3.97), with all *p* < 0.05. Group D modified-delayed *T*_arr_ scores were comparable to Group C modified-normal *T*_arr_ and significantly higher than Group B conventional–delayed *T*_arr_, reflecting improved vessel visualization under the modified protocol. Additional patient-level analysis using the mean segment score per patient yielded findings consistent with the segment-level analysis. Group B demonstrated significantly lower mean patient-level scores than Group A (3.82 ± 0.18 vs 3.97 ± 0.91, *p* < 0.001), whereas Group D showed improved scores comparable to those of Group C. Fig. 4 illustrates these findings, demonstrating that the modified protocol provides clearer visualization of distal coronary branches compared with the conventional protocol. Improved visualization was particularly evident in distal coronary branches, which demonstrated better contrast conspicuity under the modified protocol. Interobserver agreement between the two radiologists was excellent across all groups (*kappa* = 0.80–0.97), confirming the reliability of the visual scoring system.

**Table 3:** Subjective image quality assessment scores across groups

Scores	Group A (conventional–normal *T*_arr_) *n* = 46	Group B (conventional–delayed *T*_arr_) *n* = 32	Group C (modified–normal *T*_arr_) *n* = 50	Group D (modified–delayed *T*_arr_) *n* = 31
Overall scores for all segments				
Score 4 (excellent)	98.11% (624/636)	86.83% (356/410)^a^	91.99% (609/662)^a^	94.24% (409/434)^b^
Score 3 (acceptable)	1.89% (12/636)	13.17% (54/410)^a^	8.01% (53/662)^a^	5.76% (25/434)^b^
Score 2/1 (non-diagnostic)	0.00% (0/636)	0.00% (0/410)	0.00% (0/662)	0.00% (0/434)
Mean segment score	3.98 ± 0.14	3.87 ± 0.34	3.92 ± 0.27	3.94 ± 0.23
*Kappa*	0.80	0.82	0.95	0.97
Mean score per patient	3.97 ± 0.91	3.82 ± 0.18^a^	3.91 ± 0.17	3.94 ± 0.10
Scores for the three main branches				
LAD	3.99 ± 0.07	3.91 ± 0.28^a^	3.95 ± 0.21^a^	3.95 ± 0.22
LCX	3.97 ± 0.17	3.81 ± 0.40^a^	3.89 ± 0.31^a^	3.88 ± 0.32
RCA	3.97 ± 0.17	3.86 ± 0.35^a^	3.92 ± 0.27^a^	3.95 ± 0.22^b^

^a^Means this parameter was significantly different from that in Group A

^b^Means this parameter was significantly different from that in Group B

### Quantitative measurement results

**Fig. 5:**
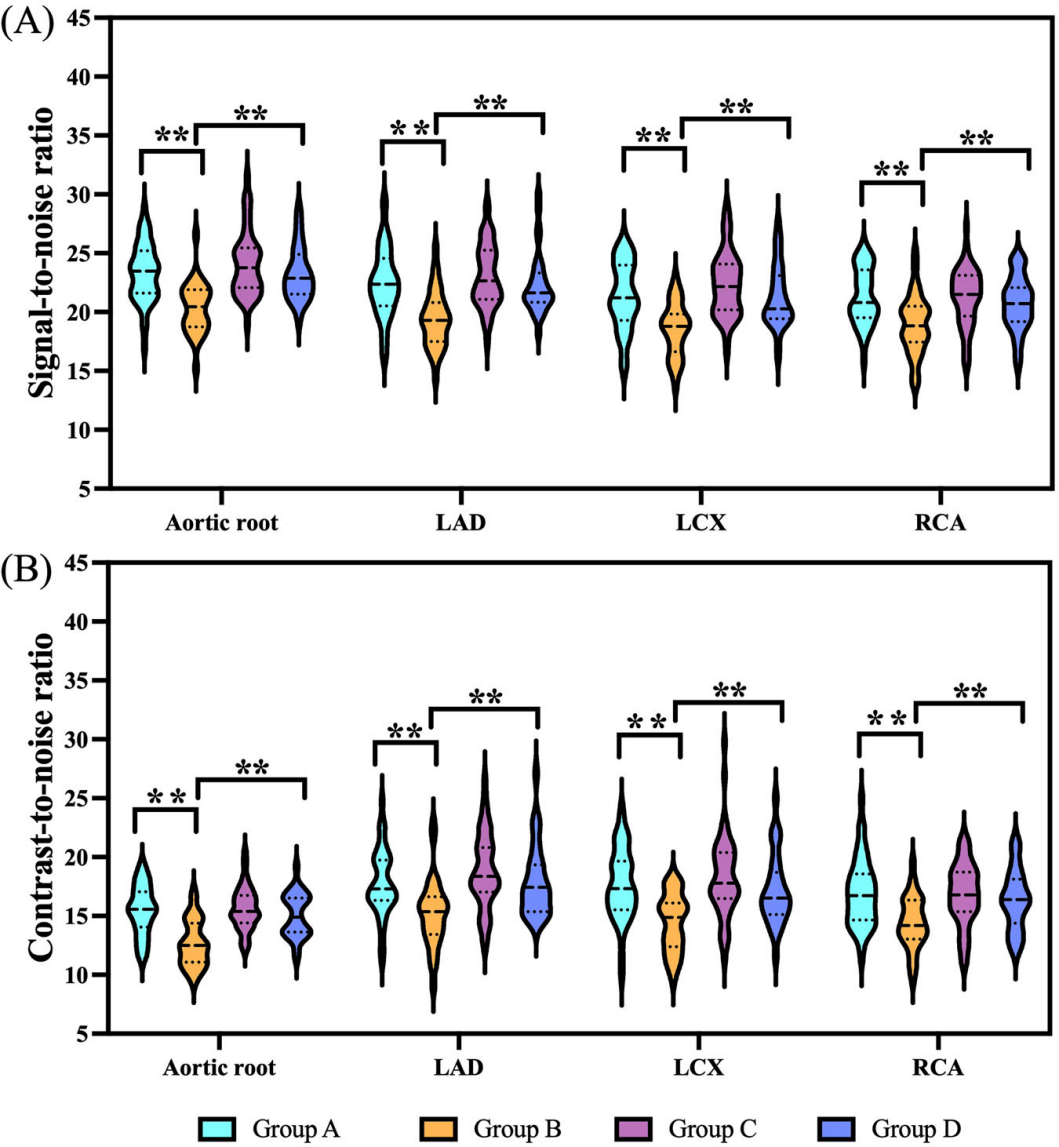
Violin plot of SNR (**A**) and CNR (**B**) measured at different sites. ^**^p < 0.001, statistically significant difference. Group A: conventional–normal *T*_arr_, Group B: conventional–delayed *T*_arr_, Group C: modified–normal *T*_arr_, and Group D: modified–delayed *T*_arr_

Quantitative measurement results, including SNR and CNR, further demonstrated the performance advantage of the modified protocol in patients with delayed contrast arrival (Table 4). Group D modified-delayed *T*_arr_ exhibited significantly higher SNR and CNR across all evaluated coronary segments compared with Group B conventional–delayed *T*_arr_ (all *p* < 0.001). For instance, SNR in the AAO was 12.64 ± 1.96 in Group B vs 15.07 ± 1.86 in Group D (*p* < 0.001). Similarly, CNR in the LAD artery was higher in Group D than in Group B (22.37 ± 2.66 vs 19.41 ± 2.40, *p* < 0.001). These results are illustrated in Fig. 5, which presents violin plots of SNR and CNR across all groups.

**Table 4:** Comparison of quantitative enhancement results

Parameters	Group A (conventional–normal *T*_arr_) *n* = 46	Group B (conventional–delayed *T*_arr_) *n* = 32	Group C (modified–normal *T*_arr_) *n* = 50	Group D (modified–delayed *T*_arr_) *n* = 31
SNR				
Aortic root	15.51 ± 2.11	12.64 ± 1.96^a^	15.66 ± 1.82	15.07 ± 1.86^b^
LAD	17.72 ± 2.84	15.20 ± 2.84^a^	18.79 ± 3.21	17.91 ± 3.02^b^
LCX	17.69 ± 3.03	14.46 ± 2.44^a^	18.25 ± 3.41	17.20 ± 3.00^b^
RCA	16.94 ± 2.86	14.42 ± 2.21^a^	16.83 ± 2.62	16.37 ± 2.56^b^
CNR				
Aortic root	23.33 ± 2.49	20.36 ± 2.16^a^	24.13 ± 2.75	23.23 ± 2.29^b^
LAD	22.47 ± 2.93	19.41 ± 2.40^a^	23.09 ± 2.68	22.37 ± 2.66^b^
LCX	21.39 ± 2.88	18.53 ± 2.29^a^	22.38 ± 2.72	21.27 ± 2.65^b^
RCA	21.34 ± 2.44	18.97 ± 2.53^a^	21.31 ± 2.46	20.76 ± 2.31^b^

^a^Means this parameter was significantly different from that in Group A

^b^Means this parameter was significantly different from that in Group B

### Enhancement uniformity results

Significant differences in contrast enhancement were observed across the four groups (Table 5). In patients with delayed contrast arrival, mean attenuation values in the AAO and coronary arteries were significantly lower when the conventional protocol was used. For example, attenuation in the AAO was 316.11 ± 41.82 HU in Group B conventional–delayed *T*_arr_ compared with 383.75 ± 43.79 HU in Group A conventional–normal *T*_arr_ (*p* < 0.001). When the modified protocol was applied in patients with delayed contrast arrival, attenuation markedly increased to 375.13 ± 33.53 HU, representing a significant improvement compared with the conventional protocol within the same subgroup (*p* < 0.001).

**Table 5:** Summary of the variability in arterial attenuation

Parameters	Group A (conventional–normal *T*_arr_) *n *= 46	Group B (conventional–delayed *T*_arr_) *n* = 32	Group C (modified–normal *T*_arr_) *n* = 50	Group D (modified–delayed *T*_arr_) *n* = 31
Attenuation values (HU)				
AAO	383.75 ± 43.79	316.11 ± 41.82^a^	387.14 ± 38.51	375.13 ± 33.53^b^
LAD	365.06 ± 52.51	296.58 ± 49.62^a^	365.62 ± 40.40	356.70 ± 40.25^b^
LCX	341.80 ± 49.30	277.88 ± 44.79^a^	350.80 ± 42.94	333.57 ± 41.82^b^
RCA	341.31 ± 44.38	286.83 ± 46.65^a^	328.86 ± 42.17	323.37 ± 43.11^b^
COVs (%)				
AAO	11.41% (43.79/383.75)	13.23%^a^ (41.82/316.11)	9.95% (38.51/387.14)	8.94%^b^ (33.53/375.13)
LAD	14.38% (52.51/365.06)	16.73%^a^ (49.62/296.58)	11.05% (40.40/365.62)	11.28%^b^ (40.25/356.70)
LCX	14.42% (49.30/341.80)	16.12%^a^ (44.79/277.88)	12.24% (42.94/350.80)	12.54%^b^ (41.82/333.57)
RCA	13.00% (44.38/341.31)	16.26%^a^ (46.65/286.83)	12.82% (42.17/328.86)	13.33%^b^ (43.11/323.37)
Scan phase				
Phase I: early	0 (0/46)	0 (0/32)	0 (0/50)	0 (0/31)
Phase II: optimal	19.57% (9/46)	9.4% (3/32)	36.00% (18/50)^b^	19.35% (6/31)
Phase III: moderate	34.78% (16/46)	25% (8/32)	34.00% (17/50)	48.39% (15/31)
Phase IV: late	45.65% (21/46)	65.60% (21/32)	30.00% (15/50)^b^	32.26% (10/31)^b^

^a^Means this parameter was significantly different from that in Group A

^b^Means this parameter was significantly different from that in Group B

The COV analysis revealed that the modified free-breathing protocol significantly reduced enhancement heterogeneity (Table 5). Across all evaluated vessels—including the AAO, LAD, LCX, and RCA-COV values were consistently lower with the modified protocol in patients with delayed contrast arrival compared with the conventional protocol (all *p* < 0.001).

**Fig. 6:**
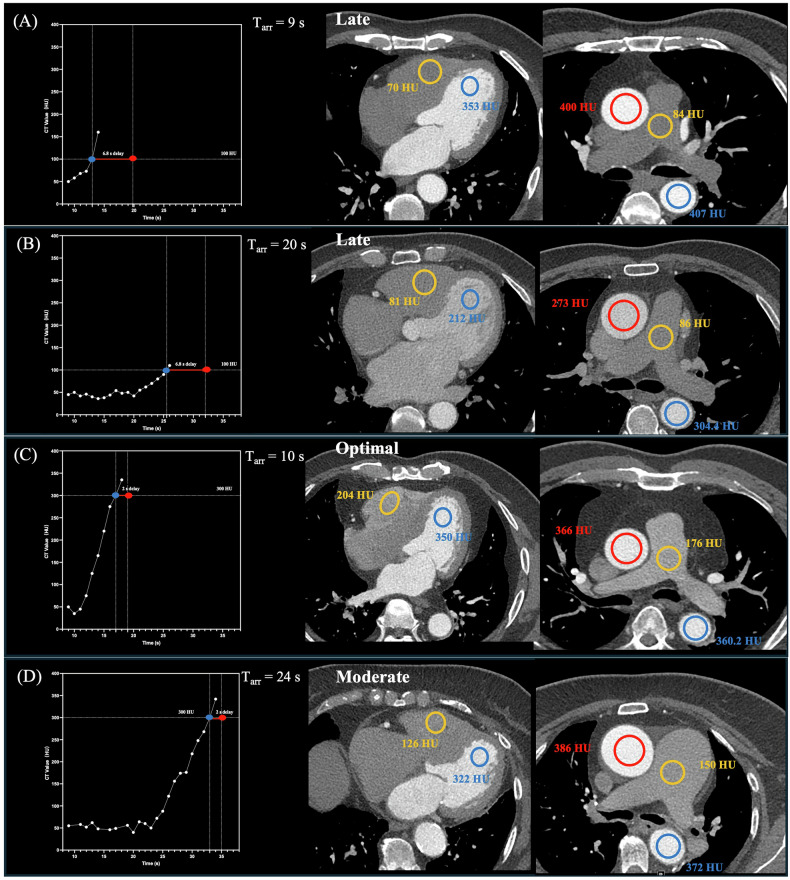
Time-attenuation curves (TACs), triggering points, scan initiation timing, and scan timing phases across the four groups. **A** Group A (conventional–normal *T*_arr_): *T*_arr_ = 9 s, Phase IV: late, attenuation_LV_ = 353 HU, attenuation_RV_ = 70 HU, attenuation_PA_ = 84 HU. **B** Group B (conventional–delayed *T*_arr_): *T*_arr_ = 20 s, Phase IV: late, attenuation_LV_ = 212 HU, attenuation_RV_ = 81 HU, attenuation_PA_ = 86 HU. **C** Group C (modified–normal *T*_arr_): *T*_arr_ = 10 s, Phase II: optimal, attenuation_LV_ = 350 HU, attenuation_RV_ = 204 HU, attenuation_PA_ = 176 HU. **D** Group D (modified–delayed *T*_arr_): *T*_arr_ = 24 s, Phase III: moderate, attenuation_LV_ = 322 HU, attenuation_RV_ = 126 HU, attenuation_PA_ = 150 HU. Blue markers indicate the triggering time points when the predefined attenuation threshold was reached, whereas red markers indicate the actual scan initiation time points. In the conventional protocol (Groups A and B), diagnostic acquisition was initiated 6.8 s after triggering, whereas in the modified protocol (Groups C and D), acquisition was initiated after a 2 s delay

Enhancement phases were evaluated based on anatomical contrast distribution (Table 5). Notably, no patients in this study demonstrated an early phase pattern (Phase I), likely reflecting the characteristics of the enrolled clinical population, in whom extremely early contrast transit patterns are uncommon. The proportion of patients achieving optimal scan phase (Phase II) was significantly higher in Group C modified-normal *T*_arr_ compared to Group B conventional–delayed *T*_arr_ (36.0% vs 9.4%, *p* = 0.007). No significant difference in Phase II distribution was observed between Group D Modified-Delayed *T*_arr_ and Group B conventional–delayed *T*_arr_. Conversely, Group B had the highest incidence of late phase (Phase IV: 65.6%), which was significantly greater than in Group C modified-normal *T*_arr_ (30.0%, *p* = 0.002) and Group D (32.3%, *p* = 0.008). These findings indicate that the free-breathing, high-threshold bolus-tracking protocol reduces the risk of late-phase acquisition in patients with delayed contrast arrival. These findings are visually supported by Fig. 6, which presents representative time–attenuation curves and scan phases from each group.

## Discussion

The current study aimed to optimize CCTA acquisition strategies in patients with delayed contrast arrival by introducing a modified bolus-tracking protocol that combines a higher triggering threshold with a shorter scan delay during free breathing. Our results indicate that, compared with the conventional breath-hold protocol, this free-breathing, high-threshold approach was associated with improved magnitude and uniformity of coronary artery enhancement when contrast arrival is delayed. In addition, overall image quality, as assessed by both visual grading and quantitative measurements, was comparable to or superior to that achieved with the conventional protocol, indicating that the modified strategy maintains diagnostic image quality while mitigating the adverse effects associated with delayed contrast timing.

Interestingly, scans acquired under delayed contrast arrival using the breath-hold protocol were associated with lower heart rates. This may reflect combined effects of breath-holding–induced autonomic modulation, including transient vagal predominance, reduced venous return, and reflex mechanisms such as the Bezold–Jarisch reflex [22, 23]. In addition, widespread β-blocker use may further attenuate sympathetic compensation, contributing to reduced heart rate responsiveness. Such hemodynamic variability may necessitate a wider ECG-gated acquisition window, potentially increasing radiation exposure. The wider window used in this study therefore represents a trade-off between radiation dose and robustness of phase selection in a heterogeneous population. The higher radiation dose observed in Group B cannot be fully explained by scan duration alone and may also relate to reduced phase-selection efficiency under less stable enhancement timing during the conventional protocol.

A key finding is that delayed contrast arrival (*T*_arr_ > 15 s) was associated with pronounced delays in coronary enhancement. Although such variability likely reflects differences in contrast circulation dynamics, it has been rarely quantified in prior CCTA studies. The modified free-breathing bolus-tracking protocol (300 HU threshold, 2 s delay) better accommodated this heterogeneity, resulting in more uniform enhancement and lower interscan variability. Compared with the conventional protocol, reduced coefficients of variation were observed in both the AAO and coronary arteries under delayed contrast conditions, accompanied by improved coronary visualization and interpretability. The increased proportion of optimal-phase acquisitions further indicates improved synchronization between coronary enhancement and scan timing, reducing late-phase imaging and heterogeneous opacification. This may translate into more consistent coronary interpretation and improved diagnostic confidence in clinical practice. This may enhance diagnostic consistency, particularly in patients with variable contrast transit. It should be noted that *T*_arr_ reflects CT-derived contrast timing rather than direct physiologic measurements [19, 21, 24, 25]. While prior studies have linked contrast timing with hemodynamics, the lack of concurrent functional assessment remains a limitation. Nevertheless, *T*_arr_ remains a practical imaging-derived marker, and future studies integrating physiologic parameters may further refine its clinical utility.

Although female participants accounted for only 3% of the study population, this reflects real-world CCTA referral patterns, where patients with suspected or established coronary artery disease are predominantly male, particularly in older populations [26, 27]. Importantly, the study focused on contrast arrival behavior and acquisition strategy rather than sex-specific differences. All examinations followed standardized protocols with uniform image quality assessment, minimizing the likelihood that sex imbalance influenced the imaging outcomes. Nevertheless, the limited representation of female patients may reduce generalizability, and further studies with more balanced sex distribution are needed.

Despite the advantages of the high-threshold strategy, potential limitations should also be considered. In patients with extremely low cardiac output or markedly delayed circulation, contrast attenuation may not reliably reach the 300 HU threshold within an acceptable monitoring period, potentially leading to delayed or failed triggering. In addition, although the post-trigger delay was shortened to 2 s, excessively prolonged contrast arrival time could still result in suboptimal acquisition timing in extreme cases. Therefore, the proposed protocol may not be universally applicable and could require further adaptation for patients with severely impaired hemodynamics.

Despite the promising results, several limitations of this study should be acknowledged. First, the subgroup sample sizes were relatively small, particularly in analyses stratified by contrast arrival timing, which may reduce statistical power and limit the detection of more subtle differences between protocols. Second, the proposed protocol relies on CT systems capable of ultra-short acquisition delays (2 s) and rapid bolus-tracking response, which may restrict its immediate applicability in institutions equipped with older or lower-performance scanners. Third, quantitative attenuation measurements were limited to proximal coronary segments. Although distal vessel visualization was evaluated subjectively using the 18-segment model, dedicated quantitative analysis of distal coronary enhancement was not performed. Finally, the present study focused primarily on image quality and enhancement characteristics rather than diagnostic accuracy. Although improved coronary enhancement and visualization may facilitate more reliable interpretation, invasive coronary angiography or outcome-based validation was not incorporated into the study design. Therefore, the direct impact of the proposed protocol on diagnostic accuracy and clinical outcomes remains to be established in future studies.

## Conclusion

This study demonstrated that the free-breathing coronary CTA protocol using a higher triggering threshold (300 HU) and an ultrashort scan delay (2 s) can improve contrast uniformity and coronary visualization in patients with delayed contrast arrival. This design minimizes timing errors by triggering the scan when sufficient enhancement is achieved, thereby reducing reliance on fixed-delay estimation. The elimination of breath-holding improves examination robustness and patient tolerance, particularly in individuals with limited cardiopulmonary reserve or inability to perform reliable breath-holds. Overall, this approach provides a more adaptive imaging strategy for patients with challenging hemodynamic conditions.

## Data Availability

All patient data in this work are not publicly available. For any inquiries or additional information, the corresponding author may be contacted.

## Abbreviations

AAO: Ascending aorta
COV: Coefficient of variation
CNR: Contrast-to-noise ratio
CCTA: Coronary computed tomography angiography
DLP: Dose length product
ED: Effective dose
IS: Interventricular septum
LAD: Left anterior descending
LCX: Left circumflex
LV: Left ventricle
PA: Pulmonary artery
RCA: Right coronary artery
RV: Right ventricle
SNR: Signal-to-noise ratio
CTD_vol_: Volume CT dose index
